# Identification of key transcriptome biomarkers based on a vital gene module associated with pathological changes in Alzheimer’s disease

**DOI:** 10.18632/aging.203017

**Published:** 2021-05-24

**Authors:** Tong Zhang, Yang Shen, Yiqing Guo, Junyan Yao

**Affiliations:** 1 Department of Anesthesiology, Shanghai General Hospital, Shanghai Jiao Tong University School of Medicine, Shanghai, China

**Keywords:** Alzheimer's disease (AD), β-amyloid (Aβ), phosphorylated tau (p-tau), gene module, transcriptome biomarker

## Abstract

Dysregulation of transcriptome expression has been reported to play an increasingly significant role in AD. In this study, we firstly identified a vital gene module associated with the accumulation of β-amyloid (Aβ) and phosphorylated tau (p-tau) using the WGCNA method. The vital module, named target module, was then employed for the identification of key transcriptome biomarkers. For coding RNA, GNA13 and GJA1 were identified as key biomarkers based on ROC analysis. As for non-coding RNA, MEG3, miR-106a-3p, and miR-24-3p were determined as key biomarkers based on analysis of a ceRNA network and ROC analysis. Experimental analyses firstly confirmed that GNA13, GJA1, and ROCK2, a downstream effector of GNA13, were all increased in 5XFAD mice, compared to littermate mice. Moreover, their expression was increased with aging in 5XFAD mice, as Aβ and p-tau pathology developed. Besides, the expression of key ncRNA biomarkers was verified to be decreased in 5XFAD mice. GSEA results indicated that GNA13 and GJA1 were respectively involved in ribosome and spliceosome dysfunction. MEG3, miR-106a-3p, and miR-24-3p were identified to be involved in MAPK pathway and PI3K-Akt pathway based on enrichment analysis. In summary, we identified several key transcriptome biomarkers, which promoted the prediction and diagnosis of AD.

## INTRODUCTION

Alzheimer’s disease (AD) is the most common form of senile dementia. According to the latest statistical data from The International AD Association, the medical expenses of AD have exceeded $240 billion worldwide [1, 2]. Unfortunately, no effective therapies can currently postpone or modify AD progression. An improved understanding of the underlying processes and risk factors associated with AD will lead to better diagnosis and treatment.

AD is a genetically heterogeneous neurodegenerative disorder caused by the cumulative impacts of sequences of genes and their interrelations [3]. Over the past decade, complex genome research has successfully identified various genetic risk factors for AD. However, the translational impacts of these findings are still limited. In addition to the genome research on AD, a trend is emerging of studying the effects of dysregulation of transcriptome on AD [4]. Transcriptome is the total complement of RNA transcripts in a cell, consisting of coding and non-coding RNAs. Transcriptome analysis could provide insights into tissue and time-dependent gene expression features, which allows investigating mechanisms associated with AD in previously unattainable details.

A range of coding RNAs (mRNAs) have been recognized as biomarkers for the diagnosis, prognosis, and therapy of AD [5–10]. On the other hand, the post-translational regulation of gene expression by non-coding RNAs (ncRNAs) has been recently recognized for their crucial roles in pathophysiological processes in AD [11–14]. Long non-coding RNAs (lncRNAs) can function as competing endogenous RNAs (ceRNAs) that sponge micro RNAs (miRNAs), thereby rescuing miRNA-targeted mRNAs [15]. An increasing number of studies have found that ceRNA networks are involved in the molecular regulatory mechanisms of AD [16, 17].

In spite of the identification of several transcriptome biomarkers and the intense attempts to develop drugs for preventing and treating AD, no effective therapies are available yet [18]. The treatment resistance of AD, resulting from the molecular complexity, demands comprehensive identification of new transcriptome biomarkers for early monitoring and therapy improvement.

β-amyloid (Aβ) and phosphorylated tau (p-tau) are thought to be hallmarks of AD pathology. The formation and accumulation of both Aβ and p-tau have been reported to increase with aging [19]. Aβ deposition-induced plaque formation is strongly associated with the disease state of AD patients. The severity of plaques is reflected by the plaque score, with higher scores indicating greater pathology. Additionally, the accumulation of p-tau can ultimately result in the formation of neurofibrillary tangles (NFTs), leading to synaptic dysfunction and neuronal loss. The BRAAK stage has been generally applied to evaluate the distribution and severity of NFTs [20].

Transgenic mice with five familial AD mutations (5XFAD - co-overexpressing amyloid precursor protein (APP) with three mutations (K670N/M671L, I716V, and V717I) and presenilins (PS1) with two mutations (M146L and L286V)) were appropriate for studies on pathological changes in AD. 5XFAD mice initially develop cerebral Aβ_42_ accumulation at 1.5 months of age, while amyloid deposits firstly appear at 2 months and increase with aging [21]. Moreover, tau protein has been reported to be more phosphorylated in 2 mo 5XFAD mice than in their wild-type littermates (LMs). With increasing age, p-tau accumulates even more [22–24].

In this study, the weighted gene co-expression network analysis (WGCNA) was conducted to identify the vital gene module related to the progression of Aβ and p-tau [25]. The target module was then employed to identify key transcriptome biomarkers, including genes and ncRNAs. Firstly, hub genes were filtered by evaluating module membership (MM) value and gene significance (GS) value. Hub ncRNAs were identified through regulatory ability evaluation in a ceRNA network constructed based on functional sub-modules extracted from the target module. Subsequently, refined hub genes and ncRNAs were further identified based on ROC analysis, and then their quantitative expression was verified in 5XFAD mice. Finally, GSEA and KEGG pathway enrichment analysis were respectively performed to explore the potential functions of these refined hub genes and ncRNAs.

## RESULTS

### WGCNA and functional enrichment analysis of gene modules

To determine if all samples in GSE29378 were appropriate for network analysis, we investigated the sample dendrogram. The results indicated that all 63 samples mainly yielded two clusters, where GSM726085, GSM726092, GSM726098, GSM726100, GSM726101, GSM726102, GSM726106, GSM726108, GSM726113, GSM726114, GSM726115, GSM726120, GSM726121, GSM726122, GSM726125, GSM726126, GSM726127, GSM726128, GSM726129, GSM726130, GSM726133, GSM726138, GSM726140, and GSM726141 became one group while the remaining samples formed the other. And there was no potential outlier in all 63 samples in GSE29378 (Supplementary Figure 4A).

We then performed a network topology analysis to explore the appropriate soft-thresholding power for WGCNA. The results suggested that when the power value was set as 7 (scale-free R^2^ = 0.85), the network possessed scale-free topology with integral modular features (Supplementary Figure 3A). By setting the cut height as 0.3, 12 modules were eventually identified (non-clustering genes were shown in gray) (Supplementary Figure 3B). Details of the 12 modules were provided in Supplementary Table 1.

To confirm the independence of each module, we conducted an interaction relation analysis. 1000 genes were randomly extracted from the 12 modules to design a network heatmap. As shown in the network heatmap (Supplementary Figure 4B), each block’s color represents the overlap degree of two corresponding genes on the horizontal axis and vertical axis. Dark color represents low overlap, and progressively lighter yellow color indicates higher overlap. The results indicated that genes from different modules had low overlap degrees, while blocks along the diagonal showed lighter colors, which indicated that each module was independent of the others. Hierarchical clustering dendrogram of the 12 module eigengenes (MEs) revealed two main clusters: one contained 3 modules (tan, blue, and yellow modules), while the other contained 9 modules (black, magenta, brown, green, purple, green-yellow, turquoise, pink, and red modules; Supplementary Figure 4C).

KEGG pathway enrichment analysis was conducted to investigate each module’s potential functions (Supplementary Figure 5). The results indicated that the “protein digestion and absorption” pathway was enriched in the black module. Genes in the blue module and brown module were enriched in the “neurodegeneration” and “valine, leucine and isoleucine degradation” pathways, respectively. “Glycine, serine and threonine metabolism” and “focal adhesion” were severally enriched in the green module and green-yellow module, while genes in the magenta module and pink module were respectively associated with the “tuberculosis” and “ribosome” pathways. The “Huntington’s disease”, “protein processing in endoplasmic reticulum”, and “neuroactive ligand-receptor interaction” pathways were respectively enriched in the purple, red, and tan modules. The turquoise module and yellow module were singly enriched in the “herpes simplex virus 1 infection” and “axon guidance” pathways. In summary, we identified 12 gene modules by performing WGCNA on dataset GSE29378 and parts of them were enriched in functional pathways associated with AD.

### Identification of the target gene module

**Figure 1:**
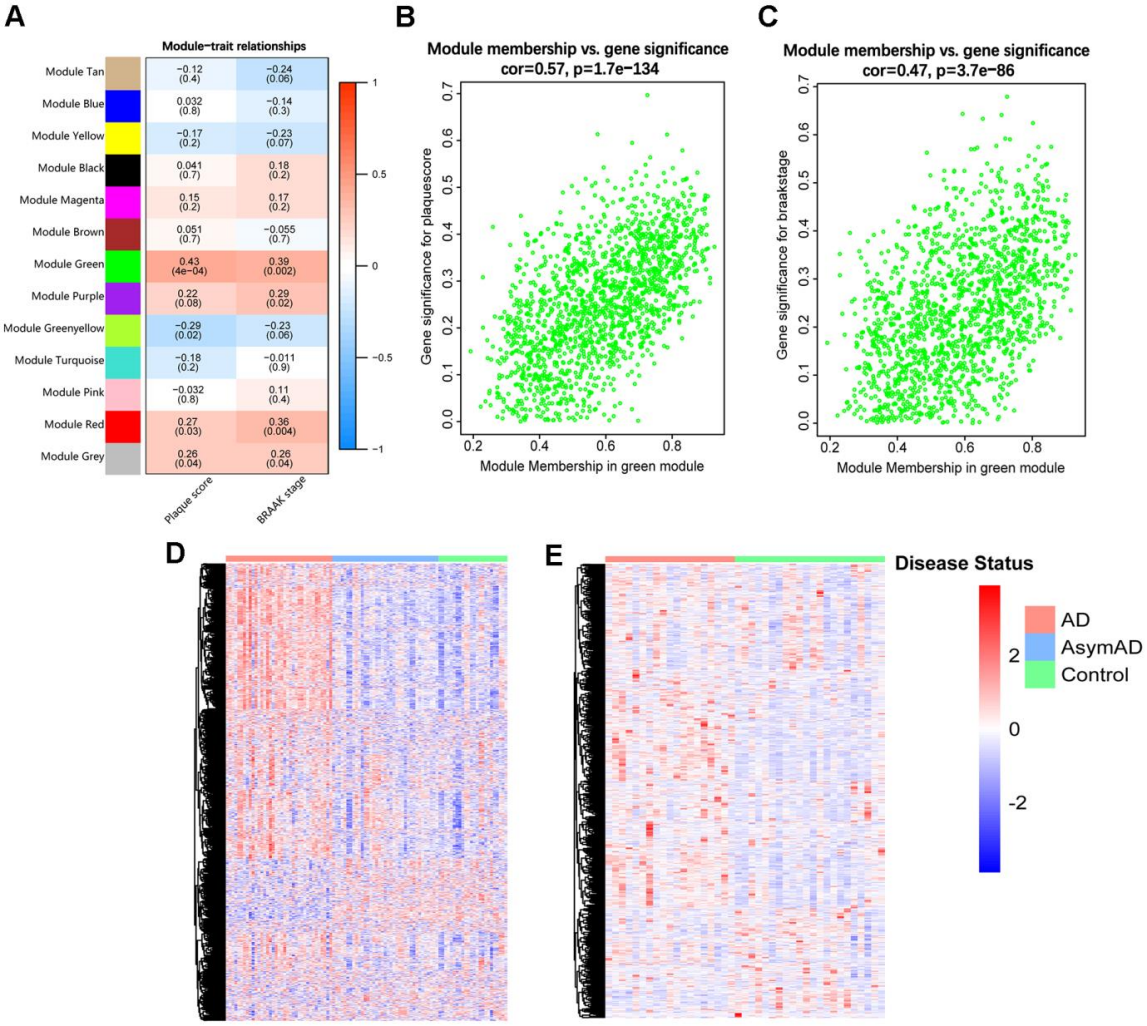
**Identification of the target module.** (**A**) Heatmap of the correlations between MEs and clinical traits of AD. Each cell contains the correlation coefficient and *p*-value (within brackets). (**B**, **C**) Scatter plots of GS value of plaque score (**B**) and BRAAK stage (**C**) vs. the MM value of each gene in the target module. (**D**) Heatmap of genes in the target module of entorhinal cortex samples in GSE118553. (**E**) Heatmap of genes in the target module of hippocampus samples in GSE48350. AsymAD, asymptomatic AD.

To identify the target module from the 12 resulting gene modules, we performed a module-trait analysis, and the analytical results were visualized in a heatmap, where the green module was detected positively correlated with both plaque score (Aβ deposition) (r = 0.43, *p* = 4×10^−4^) and BRAAK stage (p-tau accumulation) (r = 0.39, *p* = 0.002) (Figure 1A). In addition, the correlation between the MM value and GS value of each gene in the green module was analyzed. The results indicated that genes in the green module showed strong relationships with both plaque score (r = 0.57, *p* =1.7e−134) and BRAAK stage (r = 0.47, *p* = 3.7e−86) (Figure 1B, 1C). Thus, the green module was chosen as the target module for further study.

We further displayed expression heatmaps of genes in the target module based on two independent AD-related datasets (GSE118553 and GSE48350). Each heatmap’s vertical axis represents “gene symbol”, and the horizontal axis means “sample identity”. The results showed that genes in the target module were capable of discriminating AD and control individuals (Figure 1D, 1E).

### Visualization of expression pattern and chromosome location of genes from the target module

**Figure 2:**
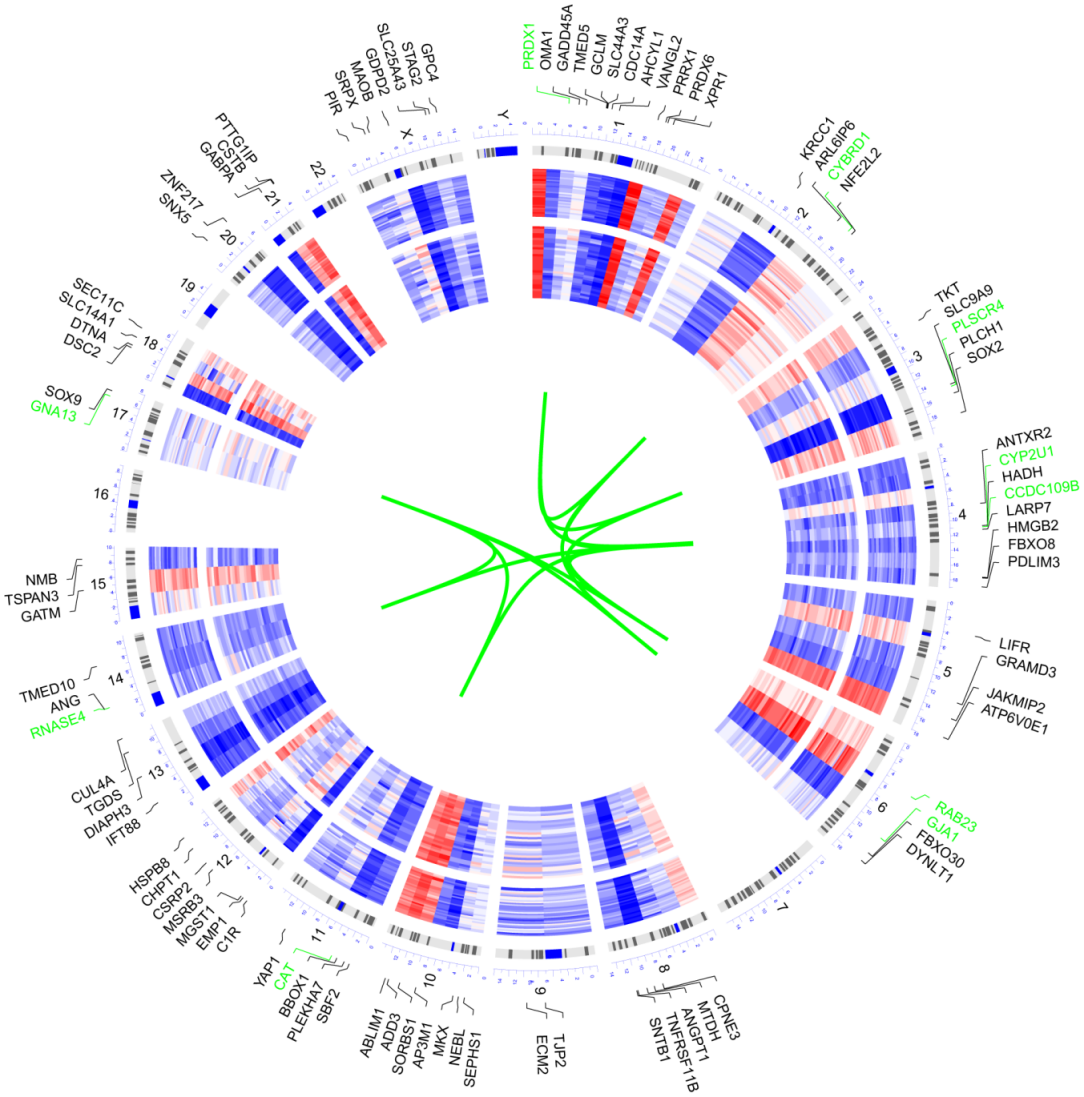
**Circular visualization of connectivity, expression patterns, and chromosomal positions of the 100 genes with the highest MM value in the target module.** The expression profiles of control (outer ring) and AD (inner ring) individuals of GSE29378 were presented in the circular heatmap. “Red” indicates upregulation, “blue” represents downregulation, and “white” denotes genes that are not present in a given dataset. The outer circle represents chromosomes; lines coming from each gene point to their specific chromosomal locations. The ten genes with the highest MM value were shown in green font and they were connected with green lines in the center of the circles.

We selected 100 genes with the highest MM value to explore the chromosome distribution and overall expression pattern of the target module (Figure 2). Among the 100 genes, the top 10 genes possessing the highest MM value, including GNA13, CYP2U1, PLSCR4, CAT, GJA1, PRDX1, CCDC109B, RNASE4, CYBRD1, and RAB23, were located on chromosomes 17, 4, 3, 11, 6, 1, 14, and 2.

### Identification of hub genes

To screen out hub genes in the target module, the criteria for selection were set as follows: MM value > 0.9, GS value to plaque score > 0.3, and GS value to BRAAK stage > 0.3. Finally, 6 hub genes were picked out, including GNA13, CYP2U1, PLSCR4, CAT, GJA1, and PRDX1. The specific information of the 6 genes was provided in Table 1.

**Table 1:** Characteristics of the hub genes.

**Gene ID**	**MM value**	**GS value (plaque score)**	**GS value (BRAAK stage)**
GNA13	0.915	0.307	0.315
CYP2U1	0.908	0.370	0.350
PLSCR4	0.908	0.481	0.417
CAT	0.907	0.369	0.343
GJA1	0.900	0.483	0.475
PRDX1	0.900	0.482	0.418

Abbreviation: MM, module membership; GS, gene significance.

### Refinement of hub genes

**Figure 3:**
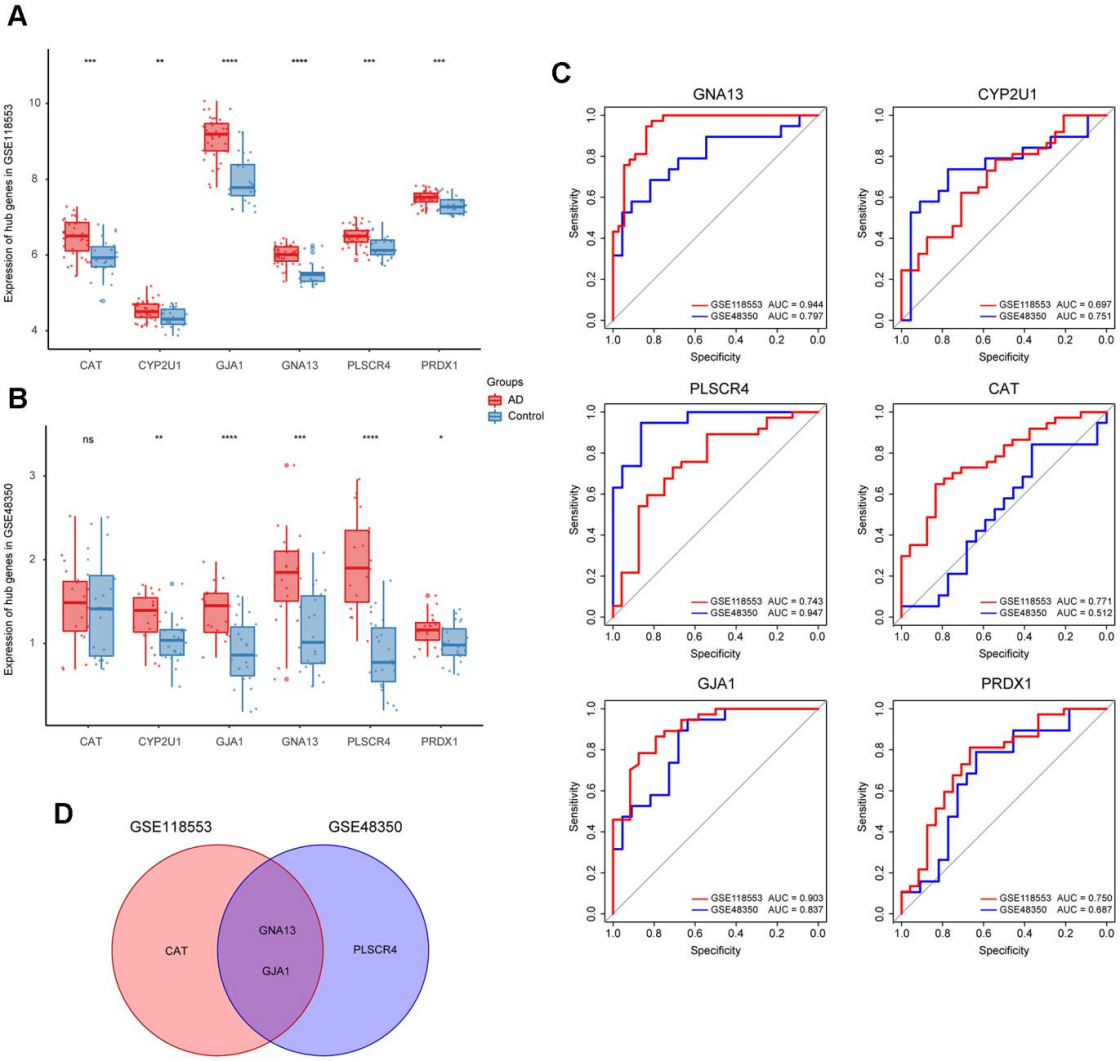
**Identification of the refined hub genes.** (**A**, **B**) Expression of the hub genes in GSE118553 (**A**) and GSE48350 (**B**). (**C**) ROC analysis of the 6 hub genes in GSE118553 and GSE48350. (**D**) The intersection of the top 3 genes with the highest AUC value in ROC analysis based on GSE118553 and GSE48350. Data were presented as the mean ± SD in each group. ns, *p* > 0.05, * *p* < 0.05, ** *p* < 0.01, *** *p* < 0.001, **** *p* < 0.0001.

To refine hub genes, we firstly explored the expression levels of those 6 hub genes in GSE118553 and GSE48350 (Figure 3A, 3B). Results showed that all hub genes were differently expressed in AD and control groups except for CAT. Further refinement was conducted through the receiver operating characteristic (ROC) curve analysis based on GSE118553 and GSE48350 (Figure 3C). The top 3 genes with the highest area under curve (AUC) value in each ROC analysis were picked out, and the intersecting genes were selected. As a result, GNA13 and GJA1 were identified as refined hub genes of the target module (Figure 3D).

### Functional sub-module analysis of the target module

**Figure 4:**
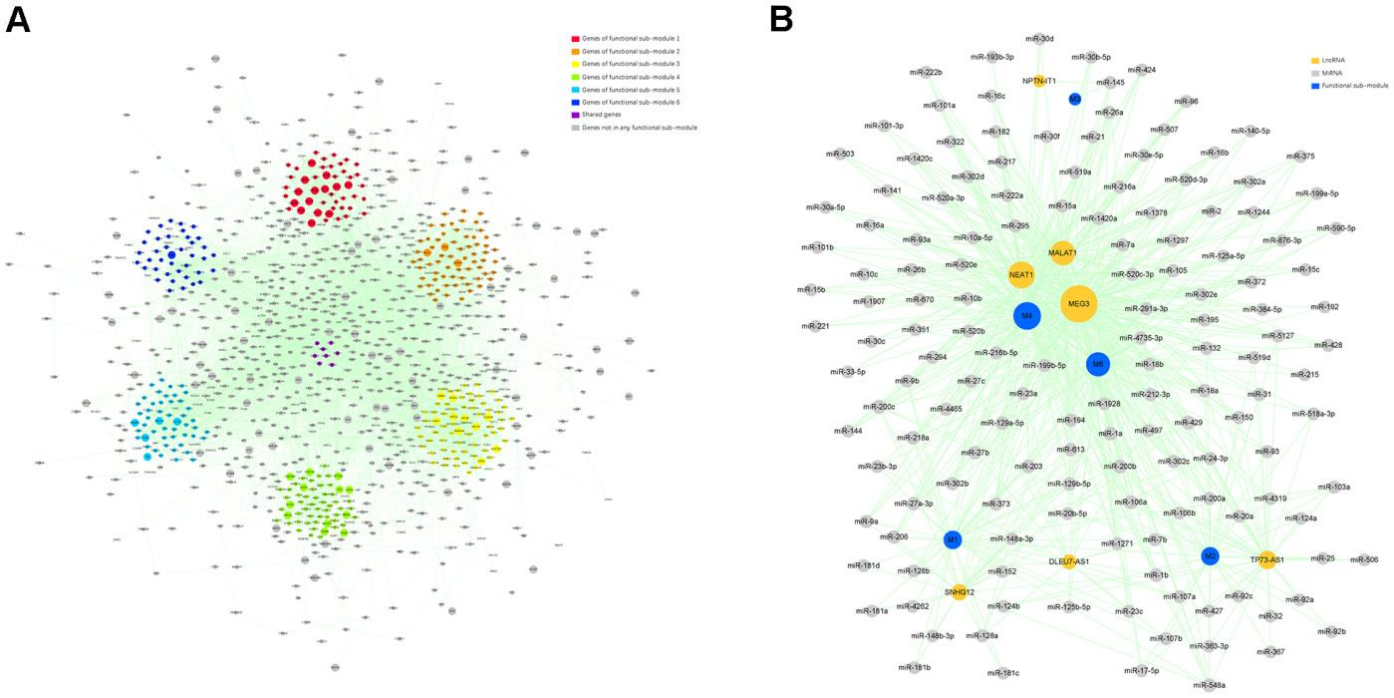
**Identification of the functional sub-modules and construction of a ceRNA network.** (**A**) The PPI network containing 6 functional sub-modules in the target module. Red nodes indicate genes in functional sub-module 1; “orange” indicates genes in functional sub- module 2; “yellow” indicates genes in functional sub-module 3; “green” indicates genes in functional sub-module 4; “bule” indicates genes in functional sub-module 5; “indigo” indicates genes in functional sub-module 6; “purple” indicates genes shared by over 2 functional sub-modules; and “gray” indicates genes not belonging to any functional sub-module. The size of certain node reflects the corresponding gene’s MM value. (**B**) A ceRNA network describing lncRNA–miRNA–functional sub-module interaction. “Indigo” indicates functional sub-module; “orange” indicates lncRNA; and “gray” indicates miRNA. The size of certain node reflects its degree value in the network.

We employed ClusterONE, a plug-in of Cytoscape, to identify functional sub-modules in the target module. Firstly, genes in the target module were employed to construct a protein-protein interaction (PPI) network using the Search Tool for the Retrieval of Interacting Genes (STRING). In the PPI network, 6693 pairs and 1147 genes were represented by edges and nodes, and the node size reflected the MM value of each gene. Six functional sub-modules containing 375 related genes were finally excavated, which were displayed in different colors in the PPI network (Figure 4A).

Then gene ontology (GO) and Kyoto encyclopedia of genes and genomes (KEGG) pathway enrichment analyses were conducted to annotate genes in functional sub-modules. Significant results with the most gene-ratio value were shown in Supplementary Figure 6. When the quantity of KEGG pathways or that of terms of each GO namespace, including biological processes (BP), molecular functions (MF), and cellular components (CC), was greater than 5, only the top 5 enriched results were displayed. GO enrichment analysis showed that genes in functional sub-module 1 (M1) were significantly enriched in “nuclear division”, “spindle”, and “protein serine/threonine phosphatase activity” terms. Genes in functional sub-module 2 (M2) were mainly enriched in “fatty acid metabolic process”, “mitochondrial matrix”, and “coenzyme binding”. The “neutrophil degranulation”, “secretory granule lumen”, and “coenzyme binding” terms were enriched in functional sub-module 3 (M3). Genes in functional sub-module 4 (M4) were enriched in “histone modification”, “transcription regulator complex”, and “transcription coactivator activity”. Genes in functional sub-module 5 (M5) were mainly enriched in “RNA splicing”, “spliceosomal complex”, and “structural constituent of the nuclear pore”. For each GO namespace, genes in functional sub-module 6 (M6) were enriched in “NF-κB signaling”, “external side of plasma membrane”, and “cysteine-type peptidase activity”. KEGG pathway enrichment analysis showed that genes in the “cell cycle” pathway were enriched in M1. Genes in M2 and M3 were enriched in the “PPAR signaling pathway” and “carbon metabolism”, respectively. The “lysine degradation” and “spliceosomes” pathways were severally enriched in M4 and M5, while genes in M6 were associated with the “NOD-like receptor signaling pathway”. Notably, these processes and pathways have been previously reported to be associated with various aspects of AD, including neuronal loss, accumulation of Aβ and p-tau, microglia dysfunction, and neurodegeneration [26–31].

### Construction of a ceRNA network and identification of hub ncRNAs

To select hub ncRNAs, we constructed a lncRNA-miRNA-functional sub-module ceRNA network. The interaction analysis of lncRNA-functional sub-module and miRNA-functional sub-module predicted 84 lncRNAs and 254 miRNAs strictly participating in 96 and 316 pairs of interplays within all 6 functional sub-modules. After a comprehensive analysis of interactions among functional sub-modules and screened ncRNAs, 5 functional sub-modules, 7 lncRNAs, and 148 miRNAs were selected for the ceRNA network construction (Figure 4B). In the ceRNA network, we found that one lncRNA, MEG3, and 22 miRNAs, which were listed in Table 2, could regulate more functional sub-modules than other ncRNAs, and they were determined as hub ncRNAs in our study.

**Table 2:** Characteristics of the hub miRNAs.

**Hub miRNA ID**	**Regulatory ability**	**Corresponding miRNA in GSE120584**	**AUC value**
miR-200b	4	miR-200b-5p/miR-200b-3p	0.537/0.567
miR-200c	4	miR-200c-5p/miR-200c-3p	0.537/0.537
miR-429	4	miR-429	0.578
miR-548a	4	miR-548a-5p/miR-548a-3p	0.585/0.589
miR-106a	3	miR-106a-5p/miR-106a-3p	0.585/0.641
miR-129a-5p	3	miR-129a-5p	0.566
miR-129b-5p	3	miR-129b-5p	0.566
miR-132	3	miR-132-5p/miR-132-3p	0.589/0.573
miR-18a	3	miR-18a-5p/miR-18a-3p	0.578/0.524
miR-18b	3	miR-18b-5p/miR-18b-3p	0.557/0.512
miR-1a	3	miR-1-5p/miR-1-3p	0.534/0.603
miR-1b	3	miR-1-5p/miR-1-3p	0.534/0.603
miR-203	3	miR-203-5p/miR-203-3p	0.532/0.552
miR-206	3	miR-206	0.497
miR-212-3p	3	miR-212-3p	0.483
miR-218a	3	miR-218a	0.579
miR-24-3p	3	miR-24-3p	0.624
miR-27a-3p	3	miR-27a-3p	0.584
miR-27b	3	miR-27b-5p/miR-27b-3p	0.587/0.552
miR-27c	3	miR-27c	0.571
miR-4735-3p	3	miR-4735-3p	0.533
miR-613	3	miR-613	0.576

Abbreviation: miRNA, microRNA; AUC, area under curve.

### Refinement of hub ncRNAs

**Figure 5:**
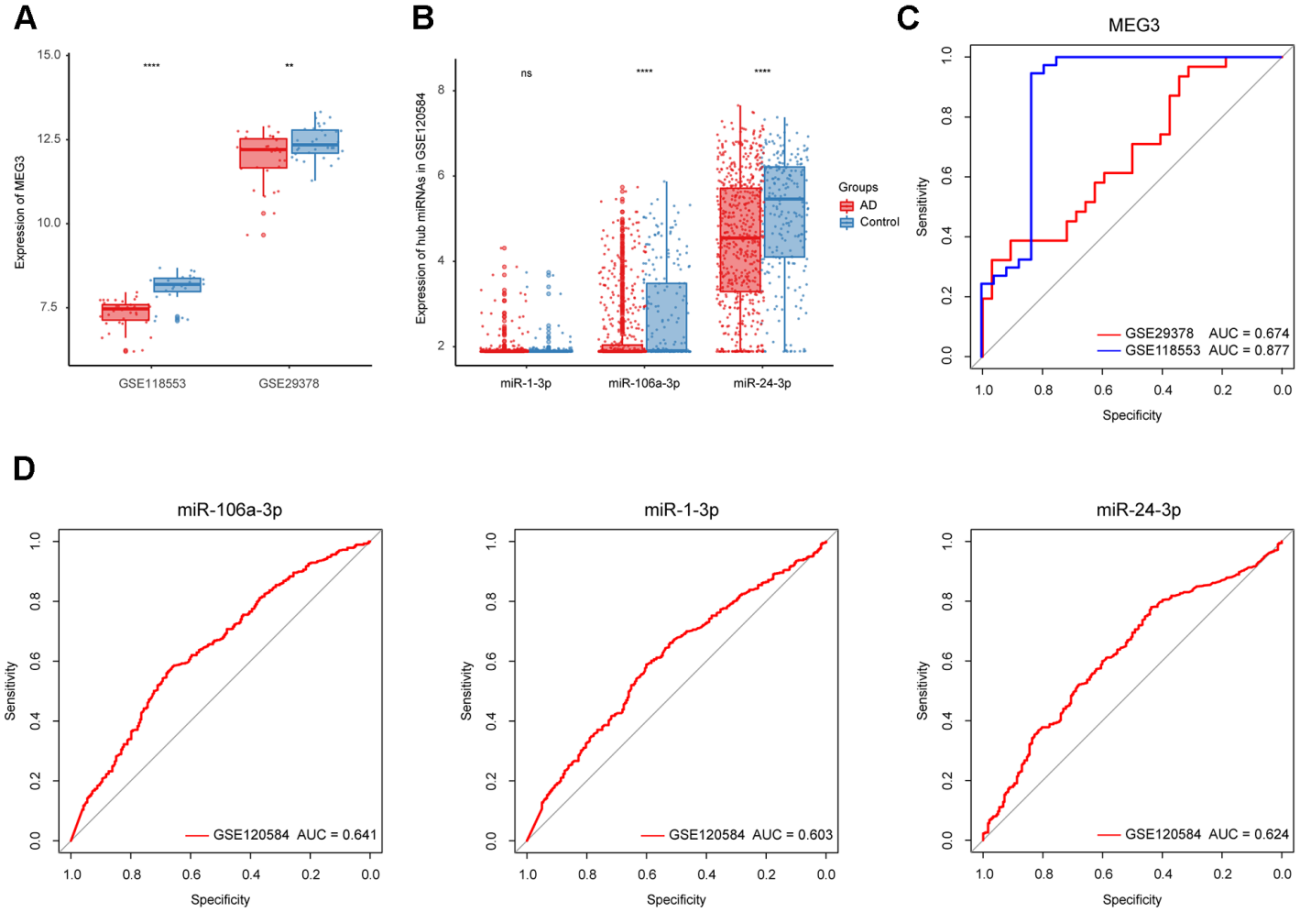
**Identification of the refined hub ncRNAs.** (**A**) Expression of MEG3 in GSE118553 and GSE29378. (**B**) Expression of hub miRNAs in GSE120584. (**C**) ROC analysis of MEG3 in GSE118553 and GSE29378. (**D**) ROC analysis of the hub miRNAs in GSE120584. Data were presented as the mean ± SD in each group. ns, *p* > 0.05, ** *p* < 0.01 and **** *p* < 0.0001.

To refine hub ncRNAs, we performed ROC analyses in three datasets, where GSE118553 and GSE29378 were employed for lncRNA refinement, and GSE120584 was used for miRNA refinement. The results showed that the AUC value of MEG3 was greater than 0.85 based on GSE118553, which indicated its potential role in AD diagnosis (Figure 5C). As for miRNA, the top 3 miRNAs with the highest AUC value, including miR-106a-3p, miR-1-3p, and miR-24-3p, were filtered from 22 hub miRNAs for further identification (Table 2 and Figure 5D). We then displayed the expression of MEG3, miR-106a-3p, miR-1-3p, and miR-24-3p in AD and control groups based on the three datasets. The results suggested that the expression of MEG3 was significantly decreased in AD patients (Figure 5A). And miR-106a-3p and miR-24-3p were differently expressed between AD and control groups, while miR-1-3p had no expression difference in the two groups (Figure 5B). Finally, MEG3, miR-106a-3p, and miR-24-3p were selected as refined hub ncRNAs in our study.

### Pathological changes in 5XFAD mice

To confirm the pathological characterization of 5XFAD and LM mice employed in this study, we detected the expression of Aβ_42_-related β-C-terminal fragment (β-CTF) and p-tau (Ser396) in hippocampus homogenates using western blotting assay. The results indicated that 4 mo 5XFAD mice had higher levels of β-CTF and p-tau (Ser396) than age-matched LM mice (*p* < 0.05) (Supplementary Figure 2A–2C). Besides, 5XFAD mice exhibited additively increased production of β-CTF and p-tau (Ser396) with aging (*p* < 0.05) (Supplementary Figure 2D–2F).

### Western blotting analysis of GNA13, ROCK2, and GJA1

**Figure 6:**
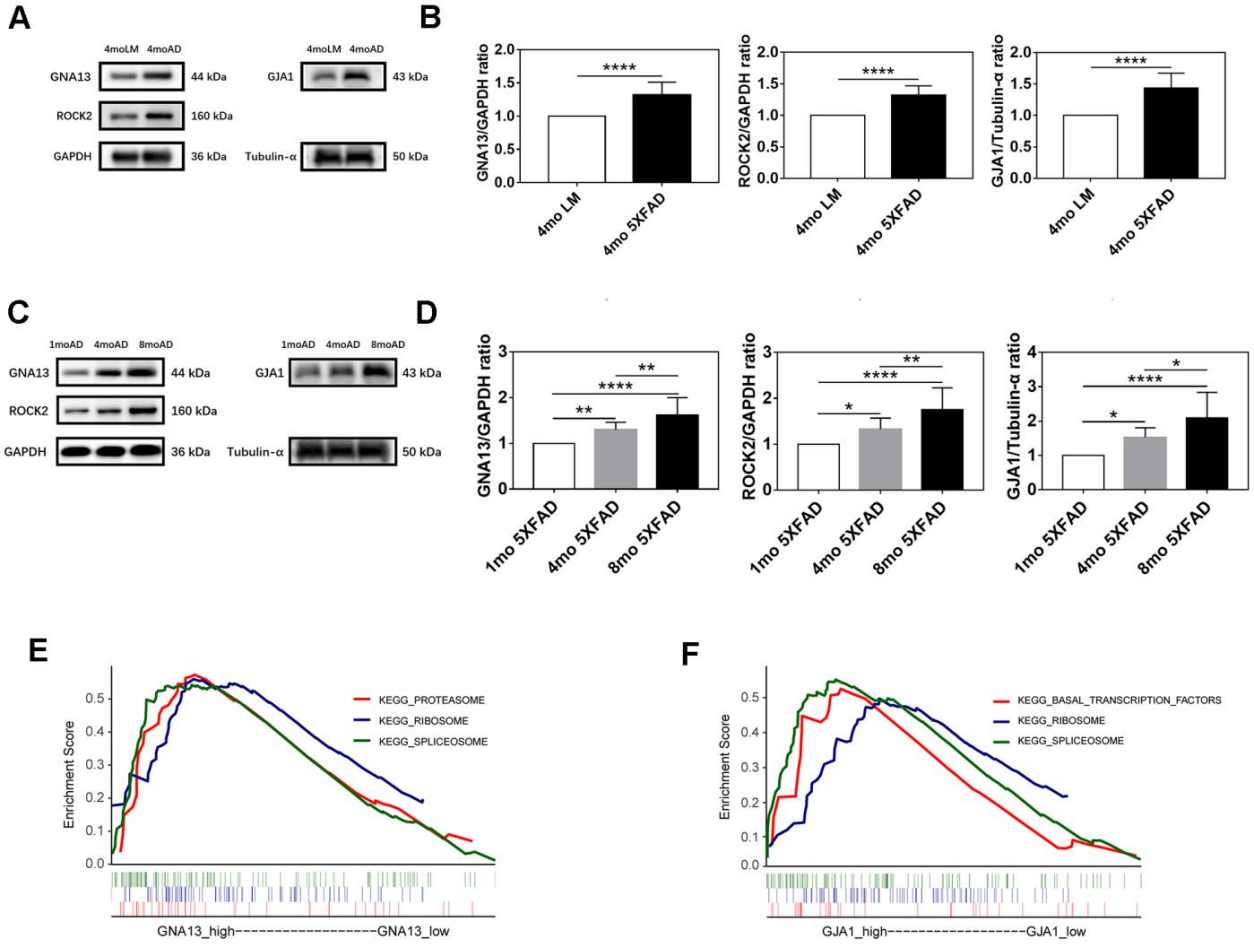
**Expression and functional analyses of GNA13, ROCK2 and GJA1.** (**A**, **B**) Representative immunoblots (**A**) and densitometry (**B**) analysis of GNA13, ROCK2, and GJA1 expression in the hippocampus of 5XFAD mice and LM mice. (**C**, **D**) Representative immunoblots (**C**) and densitometry (**D**) analysis of GNA13, ROCK2, and GJA1 expression in the hippocampus of 5XFAD mice of various ages. (**E**) Top 3 gene sets (according to NES) enriched in the high-expression group of GNA13. (**F**) Top 3 gene sets (according to NES) enriched in the high-expression group of GJA1. Data were presented as the mean ± SD of five mice in each group. * *p* < 0.05, ** *p* < 0.01 and **** *p* < 0.0001.

The western blotting analysis was applied to confirm the expression difference of GNA13, ROCK2, and GJA1 between AD and control groups. Consequently, compared with LM mice, GNA13, ROCK2, and GJA1 were all upregulated in the hippocampus of 5XFAD mice (*p* < 0.05) (Figure 6A, 6B).

To further verify the association between GNA13, ROCK2, GJA1, and aging in 5XFAD mice. We examined the expression of GNA13, ROCK2, and GJA1 in 1 mo, 4 mo, and 8 mo 5XFAD mice. As expected, the results indicated that compared with 1 mo 5XFAD mice, the expression of GNA13, ROCK2, and GJA1 was higher in the 4 mo and 8 mo groups, while 8 mo 5XFAD mice had the highest expression of all three genes (*p* < 0.05), which suggested that GNA13, ROCK2, and GJA1 were significantly upregulated in 5XFAD mice with the increase of age (Figure 6C, 6D).

### Quantitative real-time PCR (qRT-PCR) analysis of MEG3, miR-106a-3p, and miR-24-3p

**Figure 7:**
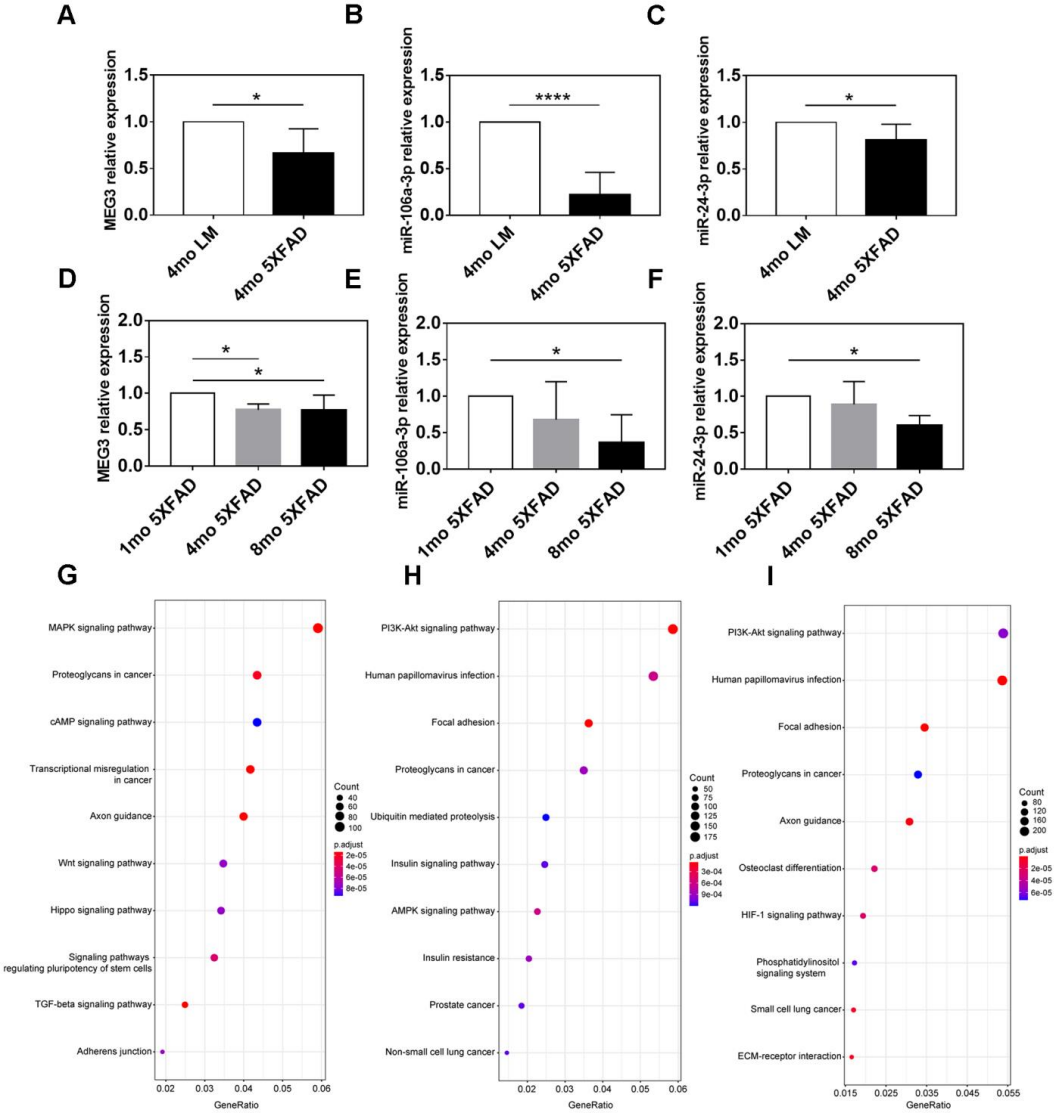
**Expression and functional analyses of MEG3, miR-106a-3p and miR-24-3p.** (**A**–**C**) The expression of MEG3, miR-106a-3p, and miR-24-3p in the hippocampus of 5XFAD mice and LM mice determined by qRT-PCR assay. (**D**–**F**) The expression of MEG3, miR-106a-3p, and miR-24-3p in the hippocampus of 5XFAD mice of various ages determined by qRT-PCR assay. (**G**–**I**) KEGG pathway analysis of target genes of MEG3, miR-106a-3p, and miR-24-3p. Data were presented as the mean ± SD of five mice in each group. * *p* < 0.05, ** *p* < 0.01 and **** *p* < 0.0001.

QRT-PCR assay was applied to confirm the expression difference of MEG3, miR-106a-3p, and miR-24-3p between AD and control groups. The results indicated that compared with LM mice, MEG3, miR-106a-3p, and miR-24-3p were all downregulated in the hippocampus of 5XFAD mice (*p* < 0.05) (Figure 7A–7C).

We further explored the association between MEG3, miR-106a-3p, miR-24-3p, and aging of 5XFAD mice. The results indicated that MEG3 was decreased in 4 mo and 8 mo groups (*p* < 0.05), compared to 1 mo group, but there was no expression difference between 4 mo group and 8 mo group (*p* > 0.05) (Figure 7D). MiR-106a-3p and miR-24-3p were both downregulated in 8 mo group, compared to 1 mo group (*p* < 0.05), however, there was no difference between 4 mo group and the other two groups (*p* > 0.05) (Figure 7E, 7F). In summary, the above results indicated that the expression of MEG3, miR-106a-3p, and miR-24-3p was partly associated with the aging of 5XFAD mice.

### Gene set enrichment analysis (GSEA) of refined hub genes

To further investigate the potential functions of GNA13 and GJA1 in AD, GSEA was performed based on GSE29378. There were 12 and 3 significant gene sets enriched in high-expression groups of GNA13 and GJA1, respectively (false discovery rate (FDR) *q* value < 0.25) (Supplementary Table 3). The top 3 gene sets with the highest normalized enrichment score (NES) were thought to reflect the potential functions of GNA13 and GJA1. The results indicated that the “proteasome”, “spliceosome”, and “ribosome” gene sets were enriched in the high-expression group of GNA13, and the “basal transcription factors”, “spliceosome”, and “ribosome” gene sets were enriched in the high-expression group of GJA1 (Figure 6E, 6F). Details of results were provided in Supplementary Table 3.

### KEGG pathway enrichment analysis of target genes of refined hub ncRNAs

We performed KEGG pathway enrichment analysis on downstream targets of the three refined hub ncRNAs to explore their functions. 4338 target genes of MEG3 were recognized based on the RAID (version 2.0) and LncRNA2Target databases (version 2.0). The target genes of miR-106a-3p and miR-24-3p were predicted based on the mirCode and mirTarBase databases, and there were 8473 and 11555 predicted targets of miR-106a-3p and miR-24-3p, respectively (data not shown). Enrichment analysis indicated that the most target genes of MEG3 were enriched in the “MAPK signaling pathway”, “proteoglycans in cancer”, and “cAMP signaling pathway”, in which the gene-ratio of the “MAPK signaling pathway” reached up to 0.059 (Figure 7G). The most targets of both miR-106a-3p and miR-24-3p were enriched in the “PI3K-Akt signaling pathway”, “human papillomavirus infection”, and “focal adhesion”. The miR-106a-3p related gene-ratio of the “PI3K-Akt signaling pathway” was 0.059, while the miR-24-3p related gene-ratio was 0.054 (Figure 7H, 7I).

## DISCUSSION

At the level of transcriptome, genetic risk factors have been demonstrated to play an increasingly essential role in the etiopathology of AD [32]. The dysregulated expression of coding RNA and non-coding RNA has been shown to be associated with the onset and progression of AD [4]. The main pathological changes in AD, including the accumulation of Aβ and p-tau, have been reported to be related to transcriptome abnormalities [32, 33]. To date, the transition in knowledge from AD-related transcriptional dysregulation to molecular mechanisms has made some progress [34]. However, the transcriptional complexity still brings the requirement for the illumination of molecular mechanisms in AD to improve the prediction of the disease in an early stage.

We employed several datasets from the Gene Expression Omnibus (GEO) database in this study. GSE29378, containing expression profiles and clinical information, is suited for the WGCNA method, while GSE118553, GSE48350, and GSE120584 were employed as independent validation datasets. GSE118553 and GSE48350 contain postmortem hippocampus and entorhinal cortex samples, respectively. The hippocampus and entorhinal cortex of AD patients suffer the accumulation of Aβ and p-tau at the early stage of the disease, which was thought to be closely associated with AD patients’ cognitive dysfunction [20, 35, 36]. GSE120584, containing 1309 serum samples, was employed for the validation of hub miRNAs.

The WGCNA method has been applied for analyzing associations among gene sets and clinical features in various diseases [25]. By using WGCNA, we recognized the green module as the target module for further analysis. The gene distribution analysis suggested that the 100 representative genes of the target module spread over almost all chromosomes, except chromosomes 7, 16, 19, 22, and Y. Chromosome 1 harbored the most genes, which indicated its potential role in influencing the pathological features of AD. After a series of bioinformatic screening analyses of the target module, two refined hub coding genes, GNA13 and GJA1, and three refined hub ncRNAs, MEG3, miR-106a-3p, and miR-24-3p, were identified for further analysis.

As for coding genes, GNA13 was found to be expressed differently in AD and control individuals in two independent datasets. The western blotting results that GNA13 was increased in 5XFAD mice further supplied the experimental evidence. To our knowledge, this is the first time that GNA13 has been found to be upregulated in AD. Then we conducted further experimental analyses to inspect whether GNA13 was associated with the progression of AD. In view that Aβ and p-tau were reported to accumulate in 5XFAD mice with aging, we employed 1 mo, 4 mo, and 8 mo 5XFAD mice for the western blotting analysis. The results that the expression of GNA13 was increased with aging in 5XFAD mice were consistent with the WGCNA results that genes in the target module were associated with the plaque score and BRAAK stage. We then performed GSEA to explore the potential functions of GNA13. The results showed that various gene expression and protein metabolism-related gene sets, including “ribosome”, “spliceosome”, and “proteasome”, were enriched in the high-expression group of GNA13, suggesting that these pathways may be involved in AD progression. ROCK2 has been previously found to be involved in the increase of Aβ in AD, however, its association with tau phosphorylation in AD has not been reported [37]. Considering the novelty of association between GNA13 and the pathological progression of AD and that ROCK2 has been found as a downstream effector of GNA13 in other diseases, we further examined the expression of ROCK2 [38, 39]. Similar to GNA13, the western blotting analysis suggested that the expression of ROCK2 was increased in 5XFAD mice. Besides, the elevated expression was associated with the aging of 5XFAD mice. The previous findings and our results provided the possibility that the dysregulation of GNA13 and ROCK2 may contribute to the pathological progression of AD in a synergistic way.

GJA1 is the other key coding gene recognized from the target module in our study. The experimental analysis gave evidence for the dysregulation of GJA1 in AD. Our western blotting results also showed that GJA1 was significantly upregulated in 5XFAD mice with the increase of age, which was consistent with previous reports that the expression of GJA1 was associated with Aβ, p-tau, and cognitive status of AD patients [40]. The GSEA results showed that the “spliceosome” gene set was enriched in the high-expression group of GJA1, which was in accordance with recent research showing that tau-mediated disruption of the spliceosome could lead to the promotion of neurodegeneration [30].

As for non-coding genes, MEG3, miR-106a-3p, and miR-24-3p achieved high AUC value in validation datasets (GSE118553, GSE29378 and GSE120584). However, the AUC values of the two miRNAs were relatively lower than MEG3, possibly because samples in GSE120584 came from serum rather than brain tissue. The qRT-PCR results that MEG3, miR-106a-3p, and miR-24-3p were significantly decreased in 5XFAD mice suggested the potentials of the three key ncRNAs as novel biomarkers of AD. Indeed, the three ncRNAs have been previously reported to be associated with AD. In detail, the upregulation of MEG3 could alleviate neuronal damage in hippocampal tissues of AD individuals [41]. The reduced expression of miR-106a in whole blood was significantly associated with an increased risk of AD [42, 43]. And miR-24 was found to reduce Aβ secretion from human cells by repressing Nicastrin expression [44]. In light of the functional sub-modules that were associated with Aβ and p-tau progression, we further explored the expression of the three ncRNAs in 1 mo, 4 mo, and 8 mo 5XFAD mice. The results showed that the expression of MEG3, miR-106a-3p, and miR-24-3p was partly associated with the aging of 5XFAD mice, possibly because their regulatory ability varied in different stages. Enrichment analysis suggested that the target genes of MEG3 were mostly enriched in the “MAPK signaling pathway”, while the targets of both miR-106a-3p and miR-24-3p were mostly enriched in the “PI3K-Akt signaling pathway”. The activation of MAPK signaling pathway has been reported to be involved in diverse AD-related events, such as tau phosphorylation and neuroinflammation [45]. On the other hand, the inhibition of PI3K-Akt signaling cascade in glial cells encompasses a central role in different cellular processes driving AD progression [46].

In summary, by combining WGCNA and other bioinformatics tools, we characterized several key transcriptome biomarkers of AD. These results may contribute to an improved understanding of the pathogenesis of AD and may lead to better diagnosis and treatment for the disease.

## MATERIALS AND METHODS

### Data collection and preprocessing

Microarray datasets, including GSE29378, GSE118553, GSE48350, and GSE120584, were collected from the GEO database. Details of each microarray dataset were provided in Table 3. GSE29378 was employed for the identification of target module, validation of MEG3, and GSEA. GSE118553 was used for validation of hub genes and MEG3. GSE48350 and GSE120584 were employed for validation of hub genes and hub miRNAs, respectively.

**Table 3:** Characteristics of the included datasets.

**Dataset ID**	**Number of samples**	**GPL ID**
GSE29378	32AD 31C (H)	GPL6947
GSE118553	37AD 24C (E)	GPL10558
GSE48350	19AD 22C (H)	GPL570
GSE120584	1021AD 288C (S)	GPL21263

Abbreviation: GSE, gene expression omnibus series; GPL, gene expression omnibus platform; AD, Alzheimer’s disease; C, control; H, hippocampus; E, entorhinal cortex; S, serum.

**Figure 8:**
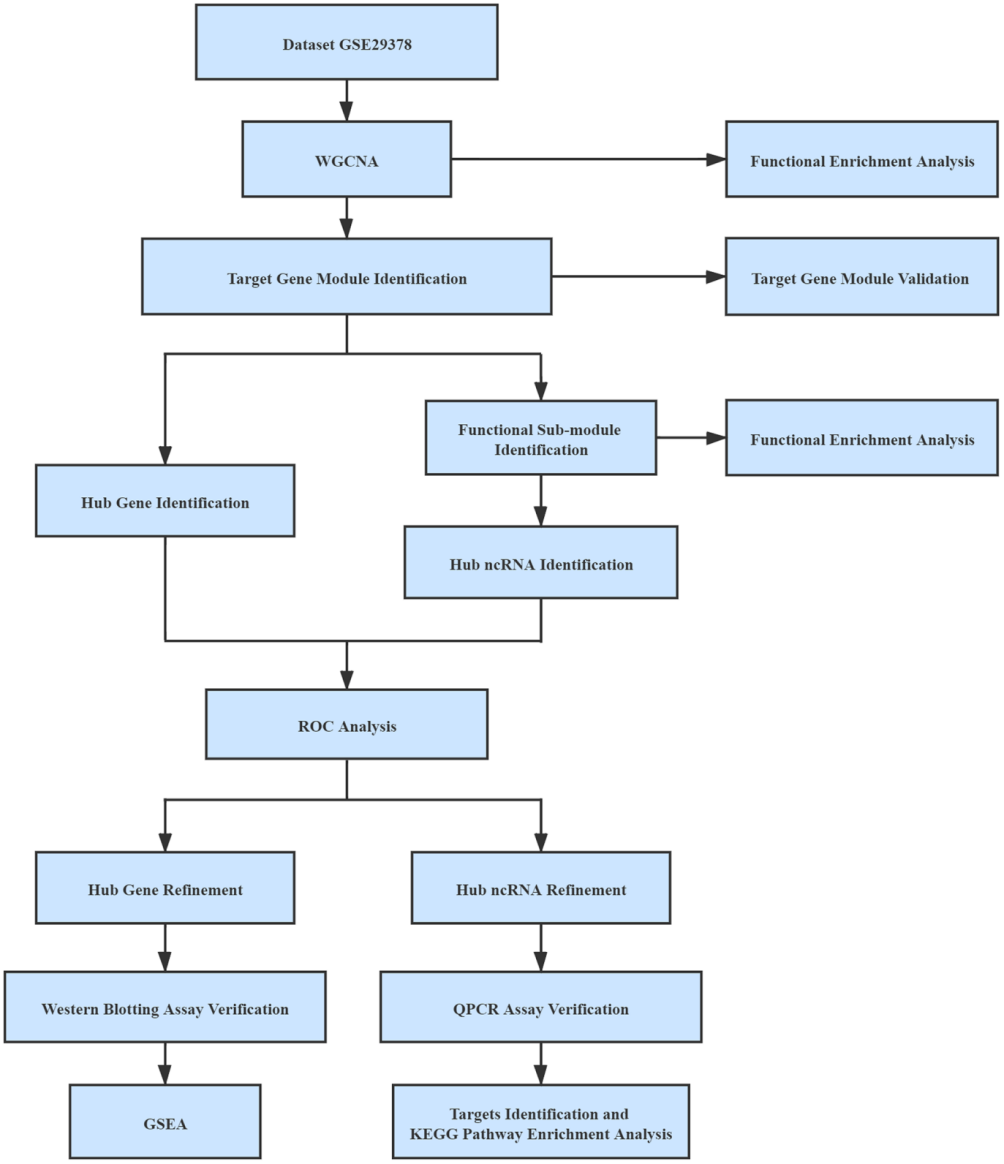
Workflow used in this study.

All expression profiles were normalized by the normalizeBetweenArrays function in the limma package in R for batch effects management [47]. In the process of mapping genes to symbols, if numerous probes were mapped to a similar symbol, their mean value was regarded as the gene manifestation value. Probes with more than one gene and empty probes were removed. The workflow used in this study was shown in Figure 8.

### WGCNA

Firstly, the goodSamplesGenes function in the WGCNA package were employed to detect samples with missing values. Legitimate samples were then delivered to the cluster analysis by the hclust function in the stats package to identify and eliminate the outliers. The WGCNA package was used to construct the co-expression network [25]. The pickSoftThreshold function was utilized to find the soft-threshold power β in accordance with standard scale-free networks. The constructed adjacency matrix was then transformed into the topological overlap matrix (TOM) with the soft-threshold power β set as 7 (scale-free R^2^ = 0.85). In addition, the dynamic tree cut method was employed, with the cut height set as 0.3 and minimal module size set as 200, to identify gene modules.

### Identification of the target module and hub genes

MEs, defined as the primary critical constituents of certain gene modules, were used to evaluate the possible relationship of gene modules with clinical traits, including plaque score and BRAAK stage. The gene module with the highest correlation index was identified as the target module. The expression heatmaps of the target module in the two independent datasets, GSE118553 and GSE48350, were displayed using the ggplot2 package in R.

The hub genes were selected based on the evaluation of MM value and GS value. MM value represents the distance from the expression profile of a gene to that of the ME, which computes how adjacent a gene is to a gene module. GS value represents the log10 transformation of the *p*-value (GS = lg *p*) in the linear regression between gene expression and AD pathological traits. In this study, hub genes were defined as genes in the target module with MM value greater than 0.9 and GS values to both plaque score and BRAAK stage greater than 0.3 [48].

### Visualization of the gene expression pattern and chromosome location

The OmicCircos package in R was utilized to visualize the expression pattern and chromosomal location of the 100 genes with the highest MM value in the target module.

### PPI network construction and functional sub-module recognition

STRING (version 11.0) online software (https://string-db.org/) was used to search the interrelationships of genes from the target module for the construction of the interaction network [49]. Cytoscape was applied for PPI network visualization [50], in which node represented protein and edge represented interaction between proteins.

Furthermore, the network was analyzed with ClusterONE, which could contribute to the classification of proteins (genes) in the network, to identify the overlapped proteins based on a cohesion algorithm. In the ClusterONE algorithm, the higher the cohesion score between two proteins is, the more likely they could produce interaction and form a protein complex [51].

### Construction of the ceRNA network

The interactions between certain lncRNA and its target genes were downloaded from RAID (version 2.0, http://rna-society.org/raid/) and LncRNA2Target database (version 2.0, http://123.59.132.21/lncrna2target/) [52, 53], the interactions between certain miRNA and its target genes were downloaded from mirCode (http://www.mircode.org) [54], and mirTarBase database (https://bio.tools/mirtarbase) [55]. And the interactions of lncRNAs and miRNAs were downloaded from mirCode database. We defined a lncRNA or miRNA as a regulator of certain functional sub-module if the genes in the functional sub-module significantly overrepresented the target genes of the lncRNA or miRNA (*p* < 0.01, hypergeometric test). Finally, the ceRNA network was constructed and visualized in Cytoscape.

### Validation of the hub genes and ncRNAs

Independent gene expression profiles containing AD hippocampus (GSE48350) and entorhinal cortex (GSE118553) samples were employed for diagnostic validation of hub genes and hub ncRNAs. ROC analyses were carried out to evaluate a certain hub gene’s sensitivity and specificity using the pROC package [56]. GSE29378 and GSE118553 were employed for the ROC analysis of hub lncRNAs. GSE120584 was employed for ROC analysis of hub miRNAs. The result of a certain ROC analysis was reflected by the corresponding AUC value.

### Animals

All progeny mice employed in this study were obtained by breeding parental male 5XFAD mice with parental female C57BL/6xSJL mice. The parental male 5XFAD mice, as described by Oakley et al., originated from Jackson Laboratory (Bar Harbor, ME, USA, Stock Number: 006554), were obtained from the agent JOINN Laboratories (Suzhou, China) [21]. The parental female C57BL/6xSJL mice were obtained from Sippr-BK laboratory animal Co. Ltd (Shanghai, China).

Genotyping of progeny mice was performed by PCR analysis of tail sample DNA, according to the supplier’s protocol. The primer details were presented in Supplementary Table 2. The results of genotyping were provided in Supplementary Figure 1, where the progeny mice with the expression of APP (377 bp) and PS1 (608 bp) were identified as 5XFAD mice, while ones with the expression of only reference DNA (324 bp) were employed as LM mice in this study.

All mice were housed in individually ventilated cages with specific pathogen-free conditions and had access to water and food pellets ad libitum. All animal experiments were in compliance with the relevant animal ethics regulations of the Animal Use Committee of Shanghai General Hospital, Shanghai Jiao Tong University School of Medicine.

### Western blotting assay

Mice were sacrificed under sevoflurane anesthesia, and the hippocampus tissues were collected and homogenized in RIPA lysis buffer (Cat# WB3100, NCM, China) with protease and phosphatase inhibitors (Cat# P002, NCM, China) for 30 min. The supernatants were harvested after centrifugation at 15000 rpm for 15 min at 4° C. The concentrations of the supernatants were measured using the bicinchoninic acid protein assay kit (Cat# NCI3225CH, Thermo, USA). After quantification, equal amounts of protein were loaded in each well and separated with 10% sodium dodecyl sulfate-polyacrylamide gel electrophoresis (SDS-PAGE). The separated proteins were electrophoretically transferred onto a polyvinylidene difluoride membrane (PVDF). The membranes were then blocked with 5% skim nonfat milk (Cat# A600669, Sangon, China) or 3% bovine serum albumin (Cat# A500023, Sangon, China) for 2h at room temperature (RT). After blocking, the membranes were incubated with primary antibody, including rabbit anti-GNA13 (44 KD, 1:1000, Cat# ab128900, Abcam, UK), rabbit anti-ROCK2 (160 KD, 1:1000, Cat# 9029S, CST, USA), rabbit anti-GJA1 (43 KD, 1:1000, Cat# ab235585, Abcam, UK), rabbit anti-Aβ_42_ (raised against a peptide corresponding to amino acids 707-713 of P05067, 17 KD, 1:1000, Cat# 700254, Invitrogen, USA), rabbit anti-p-tau (Ser396) (79 KD, 1:1000, Cat# ab109390, Abcam, UK), rabbit anti-GAPDH (36 KD, 1:2000, Cat# AF7021, Affinity, China), and rabbit anti-Tubulin-α (50 KD, 1:2000, Cat# AF7010, Affinity, China), at 4° C overnight. The membranes were then incubated with anti-rabbit secondary antibodies (1:4000, Cat# 111-035-003, Jackson, USA) at RT for 1 h. The protein bands were detected with enhanced chemiluminescence (ECL) western blot kit (Cat# P10100, NCM, China) and visualized using a ChemiDoc XRS System with Image Lab software (Bio-Rad, USA).

### QRT-PCR assay

Total RNA was extracted from hippocampus tissues by TRIzol (Cat# 15596026, Invitrogen, USA) following the manual. The RNA quality was evaluated via the A260/A280 ratio. For quantification of MEG3, cDNA was synthesized from total RNA by reverse transcriptase using the random primer (Cat# RR037A, Takara, Japan). For miR-106a-3p and miR-24-3p, RNA was reversely transcribed to cDNA using the corresponding miRNA stem-loop RT primer (Supplementary Table 4). Each sample was prepared in triplicate for a total reaction volume of 20 μl, with 250 nM forward and reverse primers, 10 μl SYBR Green (Cat# Q204, NovaBio, China), and 20 ng cDNA.

Details of the primer sequence were provided in Supplementary Table 5. All reactions were carried out in a QuantStudio 3 Real-Time PCR system. The expression of MEG3 was normalized to GAPDH, while the expression of miR-106a-3p and miR-24-3p was normalized to U6.

### GSEA

GSEA software (version 4.0.3) was used to perform GSEA [57]. Based on the median expression of certain hub genes, 31 AD samples were divided into high-expression and low-expression groups. FDR *q* value < 0.25 was regarded as statistically significant. The reference gene set, “c2.cp.kegg.v7.1.symbols.gmt”, was downloaded from the Molecular Signature Database (MSigDB, http://software.broadinstitute.org/gsea/msigdb/index.jsp).

### Functional enrichment analysis

GO and KEGG pathway enrichment analyses were conducted with a criterion of adjust-*p*-value < 0.05, using the clusterProfiler package in R [58].

### Data analysis

All data were expressed as the mean with standard deviation (mean ± SD). The statistical analysis was performed using Prism (Version 8.0) Software. The results of western blotting and qRT-PCR analyses were analyzed based on the student 2-tailed unpaired t-test and one way analysis of variance (ANOVA), followed by Tukey post hoc test. Statistical significance was considered to occur at *p* < 0.05.

## Supplementary Material

Supplementary Figures

Supplementary Tables
