# Supplementary material for: Identification of key transcriptome biomarkers based on a vital gene module associated with pathological changes in Alzheimer’s disease
Source: Aging (Albany NY). 2021 May 24;13(11):14940–67. doi: 10.18632/aging.203017 (PMC8221319; doi:10.18632/aging.203017)
Supplement: Supplementary Figures [file aging-13-203017-s001.pdf]

SUPPLEMENTARY FIGURES

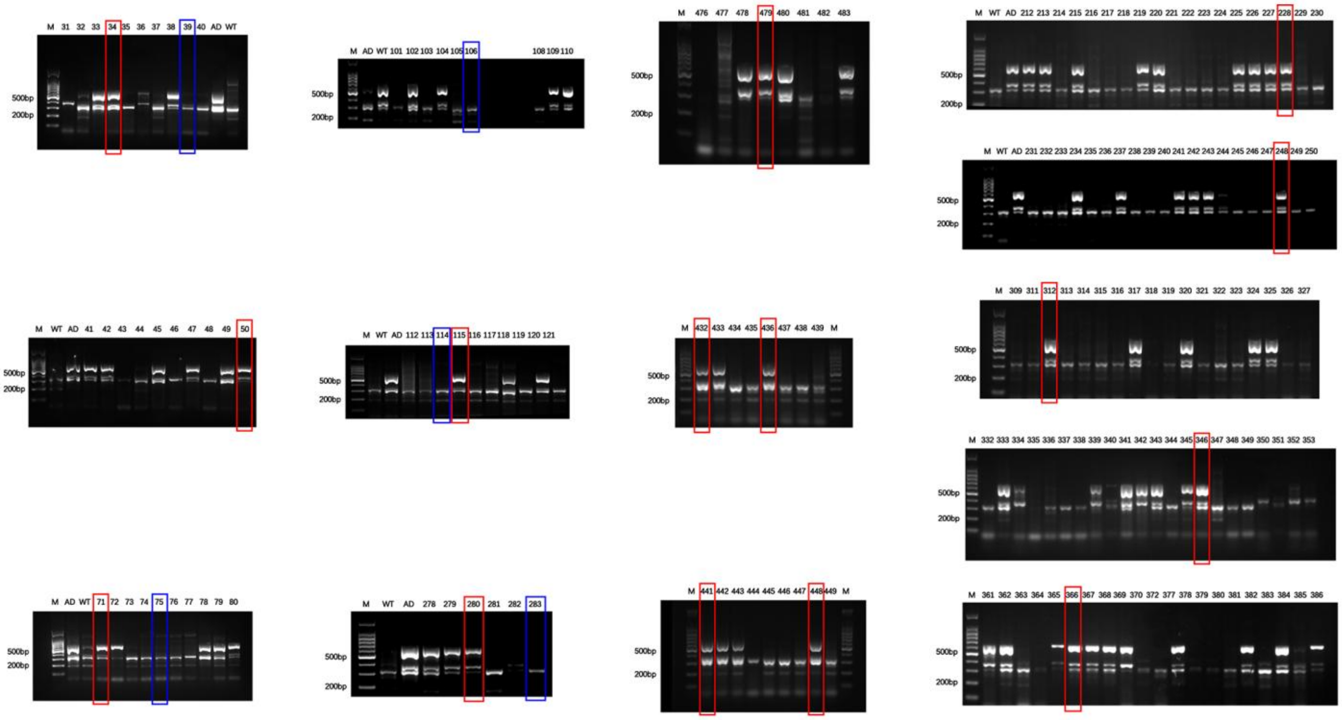

Supplementary Figure 1. Genotyping results. Genotyping results of 4 mo LM mice, 1 mo 5XFAD mice, 4 mo 5XFAD mice, and 8 mo 5XFAD mice. “M” represents a DNA molecular weight marker; “WT” represents a reference of wild-type mice; and “AD” represents a reference of 5XFAD mice. Each number on the top is the ear tag number of different mice. DNA bands enclosed by blue and red boxes represent certain mice employed in this study. Bands enclosed by blue boxes represent 4 mo LM mice, including number 39, 75, 106, 114, and 283. Bands enclosed by red boxes represent 5XFAD mice with different ages, where number 432, 436, 441, 448, and 479 are 1 mo 5XFAD mice, number 34, 50, 71, 115, and 280 are 4 mo 5XFAD mice, and number 228, 248, 312, 346, and 366 are 8 mo 5XFAD mice.

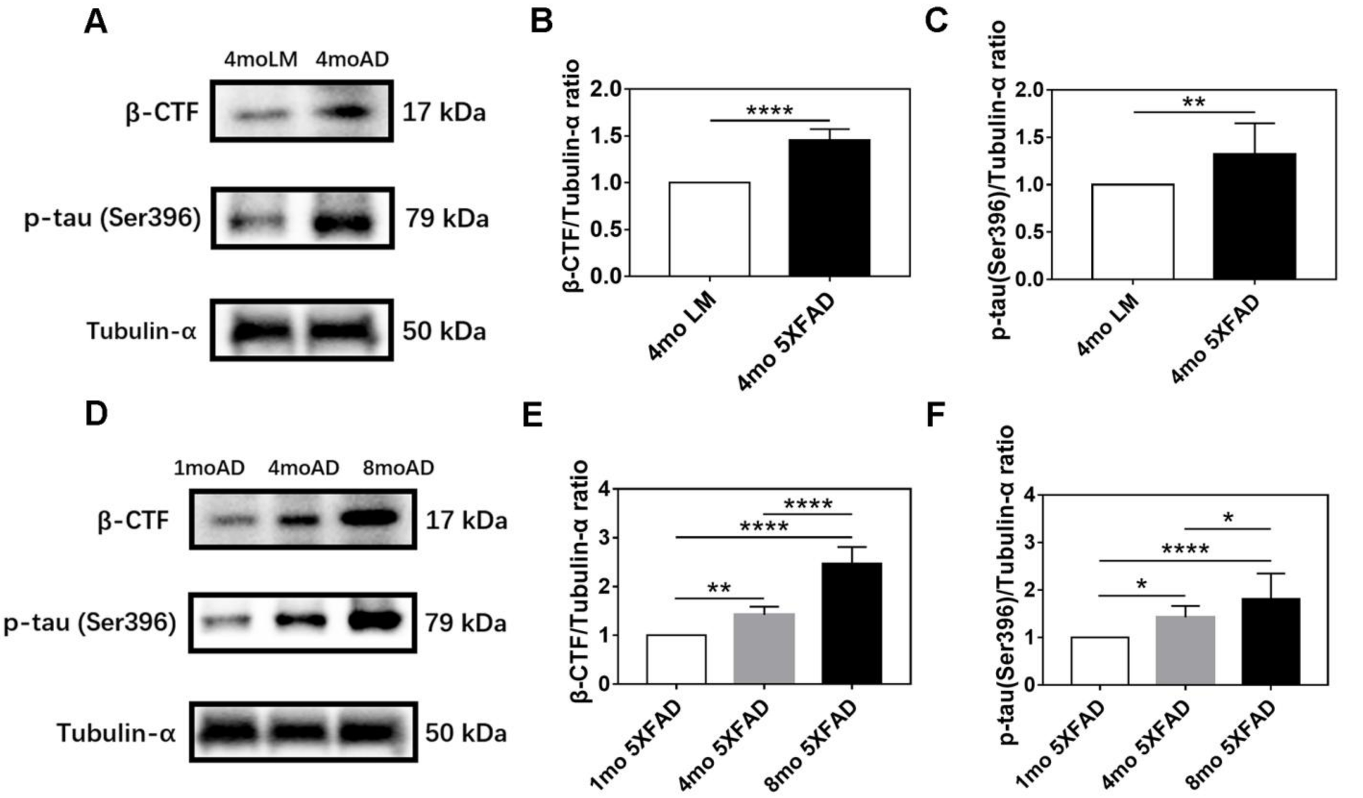

Supplementary Figure 2. Expression analysis of Aβ$_{42}$-related β-CTF and p-tau (Ser396). (**A**–**C**) Representative immunoblots and densitometry analysis of β-CTF and p-tau (Ser396) in the hippocampus of 5XFAD mice and LM mice. (**D**–**F**) Representative immunoblots and densitometry analysis of β-CTF and p-tau (Ser396) in the hippocampus of 5XFAD mice of various ages. Data were presented as the mean ± SD of five mice in each group. * $p < 0.05$, ** $p < 0.01$ and **** $p < 0.0001$.

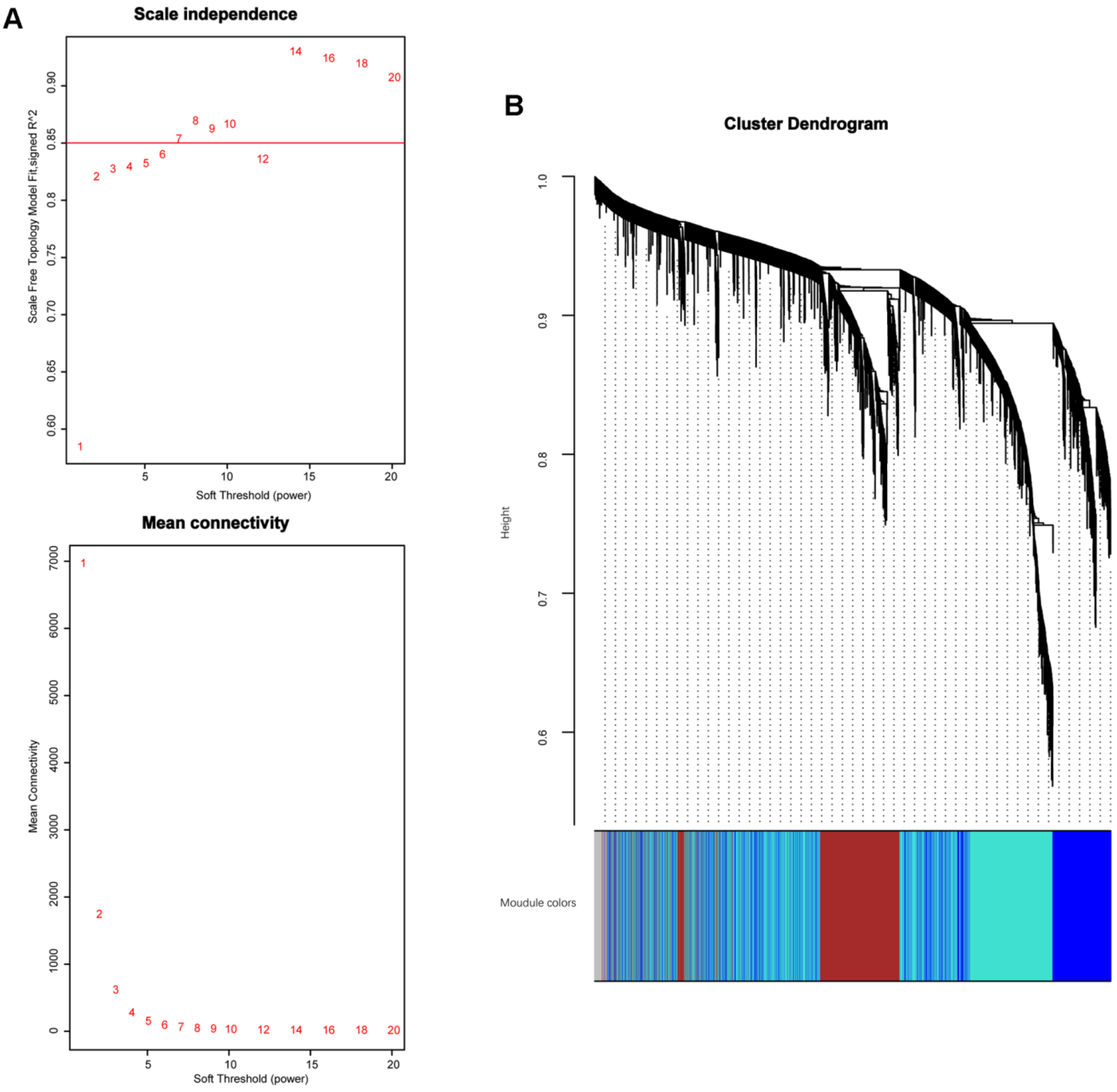

Supplementary Figure 3. Determination of the soft-thresholding power in WGCNA. (**A**) Top: analysis of the scale-free fit index; bottom: mean connectivity for various soft-thresholding powers. (**B**) Dendrogram of all genes clustered based on a dissimilarity measure (1-TOM). TOM, topological overlap matrix.

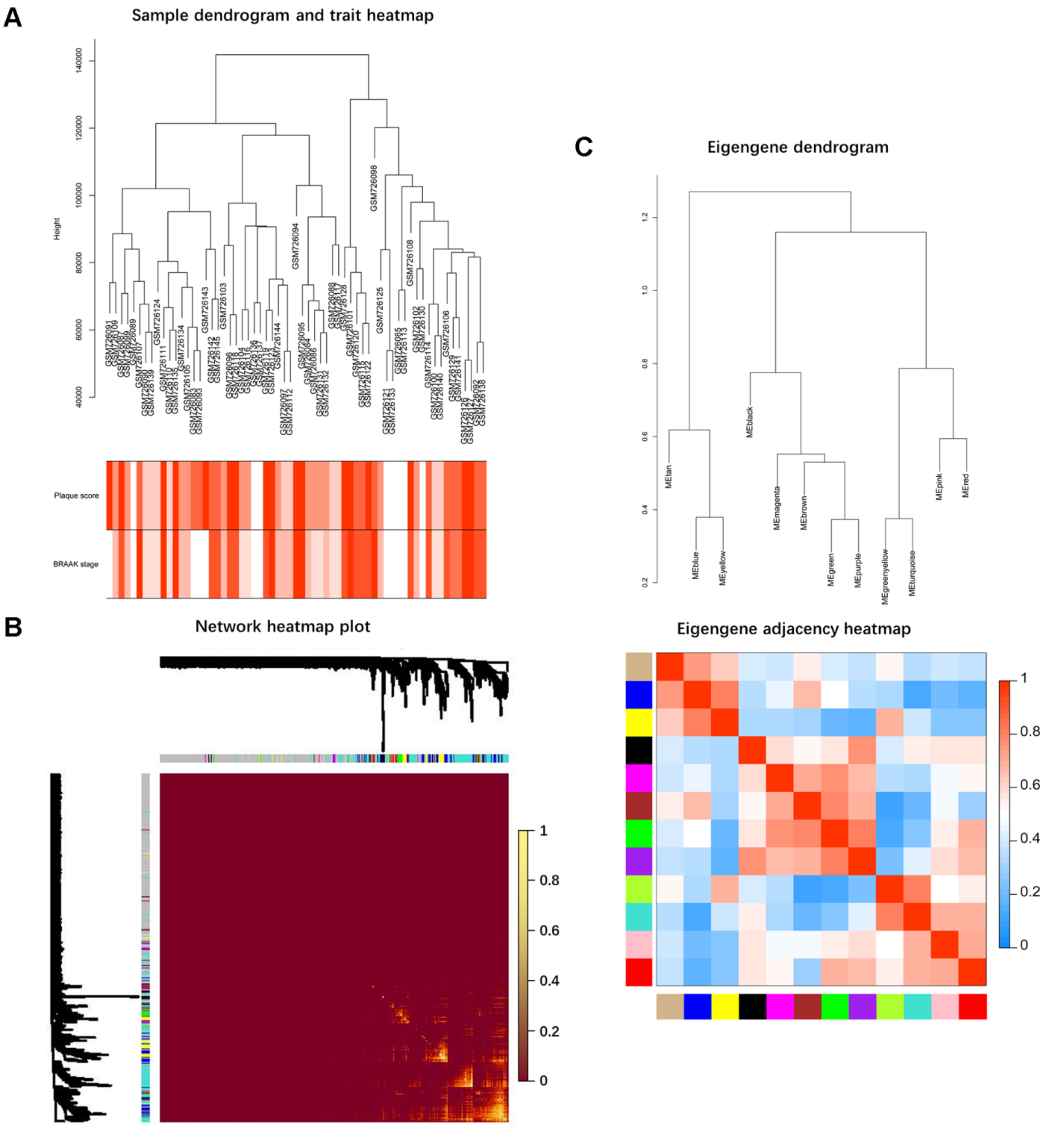

Supplementary Figure 4. Clustering of samples and identification of gene modules. (**A**) Sample clustering based on GSE29378. Color intensity varies positively with plaque score and BRAAK stage. (**B**) Heatmap of the correlations among the 12 modules. Dark color represents low overlap and progressively lighter yellow color indicates higher overlap. The gene dendrogram and module assignment were shown along the left side and the top. (**C**) Top: clustering of MEs acquired by WGCNA; bottom: heatmap plot of the correlations among eigengene adjacencies of modules.

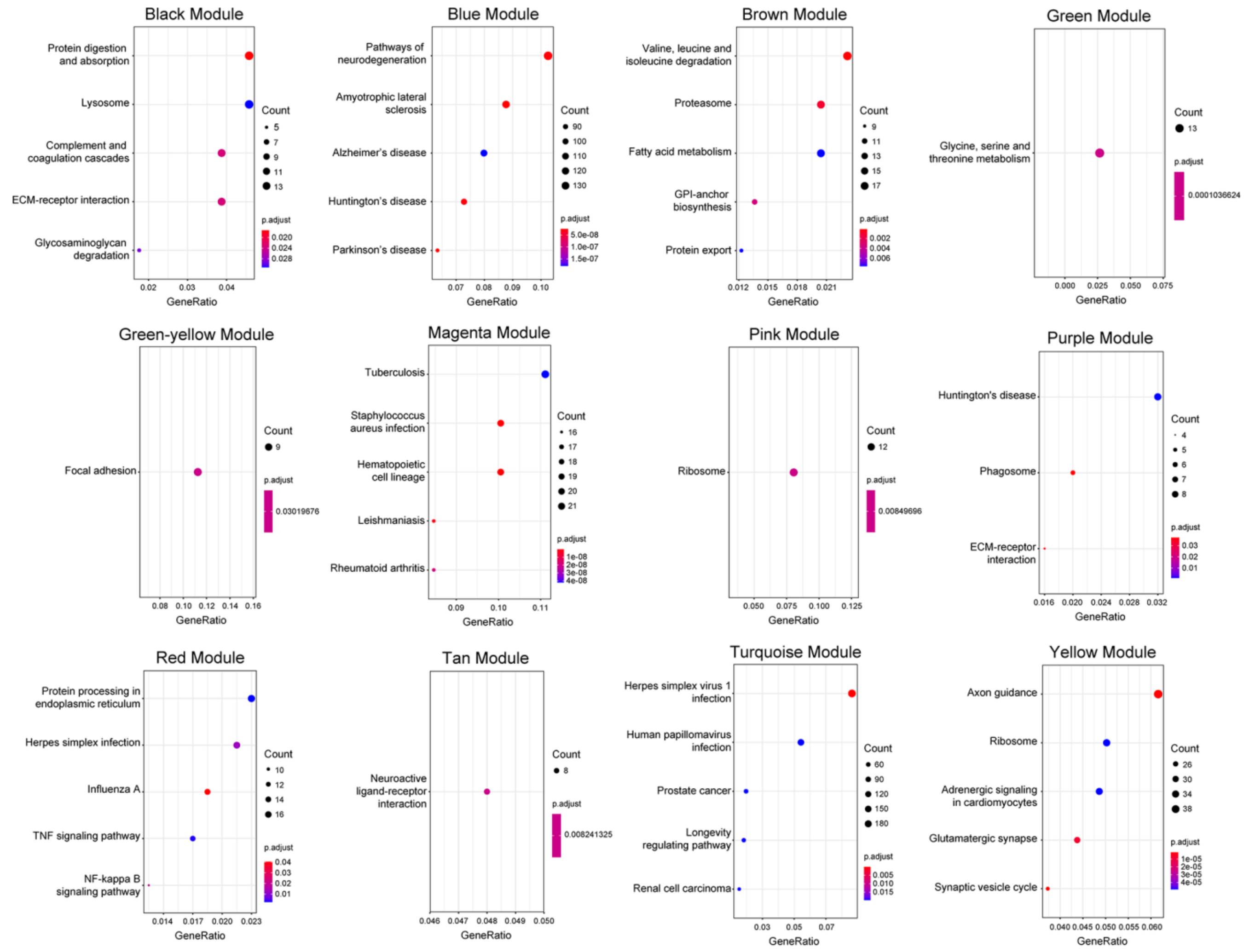

Supplementary Figure 5. Functional enrichment analysis of the gene modules. KEGG pathway enrichment results of the 12 gene modules.

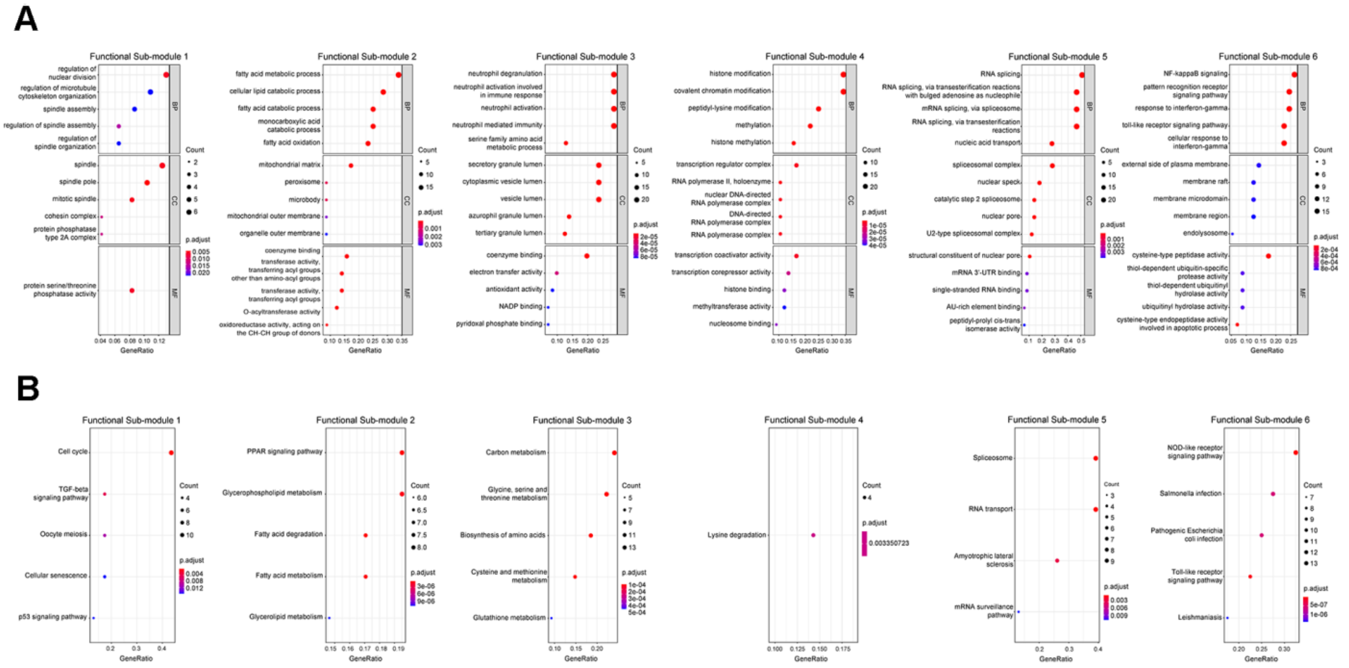

Supplementary Figure 6. GO and KEGG pathway enrichment analyses of the functional sub-modules. (**A**) GO enrichment results of the functional sub-modules. (**B**) KEGG pathway enrichment results of the functional sub-modules.
