# Supplementary material for: Identification of key transcriptome biomarkers based on a vital gene module associated with pathological changes in Alzheimer’s disease
Source: Aging (Albany NY). 2021 May 24;13(11):14940–67. doi: 10.18632/aging.203017 (PMC8221319; doi:10.18632/aging.203017)
Supplement: Supplementary Tables [file aging-13-203017-s002.pdf]

SUPPLEMENTARY TABLES

Supplementary Table 1. Size of the gene modules.

Gene module	Size (Gene number)
Module Black	828
Module Blue	3583
Module Brown	1973
Module Green	1282
Module Green-yellow	247
Module Magenta	454
Module Pink	509
Module Purple	374
Module Red	890
Module Tan	194
Module Turquoise	6543
Module Yellow	1681

Supplementary Table 2. Primer sequence of genotyping.

Prime type	Forward	Reverse
APP	AGGACTGACCACTCGACCAG	CGGGGGTCTAGTTCTGCAT
PS1	AATAGAGAACGGCAGGAGCA	GCCATGAGGGCACTAATCAT
Reference	CTAGGCCACAGAATTGAAAGATCT	GTAGGTGGAAATTCTAGCATCATCC

Supplementary Table 3. Characteristics of GSEA results.

Gene	Pathway	ES	NES	NOM p-val	FDR q-val
GNA13	Ribosome	0.567	1.875	0.008	0.009
	Proteasome	0.580	1.736	0.000	0.064
	Spliceosome	0.549	1.674	0.024	0.087
	Parkinson's disease	0.587	1.613	0.027	0.148
	Nucleotide excision repair	0.604	1.588	0.004	0.164
	RNA degradation	0.494	1.549	0.025	0.201
	Basal transcription factors	0.531	1.535	0.021	0.200
	Glycine serine and threonine metabolism	0.574	1.520	0.036	0.204
	Protein export	0.689	1.502	0.047	0.218
	Glyoxylate and dicarboxylate metabolism	0.626	1.481	0.018	0.239
	Mismatch repair	0.626	1.472	0.047	0.237
	Cell cycle	0.398	1.469	0.024	0.224
GJA1	Spliceosome	0.569	1.739	0.012	0.080
	Ribosome	0.514	1.717	0.054	0.059
	Basal transcription factors	0.542	1.579	0.017	0.231

Abbreviation: ES, enrichment score; NES, normalized enrichment score; NOM p-val, normalized p value; FDR q-val, false discovery rate q value.

Supplementary Table 4. Primer sequence of reverse transcription.

RNA	Reverse transcription
miR-106a-3p	GTCGTATCCAGTGCAGGGTCCGAGGTATTCGCACTGGATACGACGTAAGA
miR-24-3p	GTCGTATCCAGTGCAGGGTCCGAGGTATTCGCACTGGATACGACCTGTTC
U6	GTCGTATCCAGTGCAGGGTCCGAGGTATTCGCACTGGATACGACAAAAATATGG

Supplementary Table 5. Primer sequence of qRT-PCR.

RNA	Forward	Reverse
MEG3	CCTGTCGCGTCTTCCTGTGC	TGGGGTCCTCAGTCTTCTTTTCT
GAPDH	AACTTTGGCATTGTGGAAGG	GGATGCAGGGATGATGTTCT
miR-106a-3p	CGACTGCAGTGCCAGCACT	AGTGCAGGGTCCGAGGTATT
miR-24-3p	GCGTGGCTCAGTTCAGCAG	AGTGCAGGGTCCGAGGTATT
U6	GCTCGCTTCGGCAGCACATATAC	AGTGCAGGGTCCGAGGTATT
